# Supplementary material for: Multivariate characterisation of morpho-biometric traits of indigenous helmeted Guinea fowl (Numida meleagris) in Nigeria
Source: PLoS One. 2022 Jun 13;17(6):e0261048. doi: 10.1371/journal.pone.0261048 (PMC9191719; doi:10.1371/journal.pone.0261048)
Supplement: S2 Data — (PDF) [file pone.0261048.s002.pdf]

CODING KEYS:

PLUMAGE COLOUR

1. Pearl
2. Lavender
3. Black
4. White
5. Brown
6. Pied

SKIN COLOUR

1. Dark
2. Pale Red

SHANK COLOUR

1. Orange
2. Black
3. Yellow
4. White
5. Peach Black
6. Pale Pink
7. Brown
8. Pale Red
9. Red
10. Pink With Black Spot
11. Black-Orange

EYE COLOUR

1. White
2. Brown
3. Pink
4. Black
5. Bluish

EARLOBE COLOUR

1. White
2. Bluish
3. Spotted
4. Whitish Brown
5. Brown
6. Pale Pink
7. White Bluish

8. Dirty White
9. Black
10. Pink
11. Purple

HELMET COLOUR
1. Purple
2. Brown
3. Black
4. Red
5. Pink

HELMET SHAPE
1. Slanted Backward
2. Single
3. Erect

WATTLE POSSESSION
1. Present
2. Absent

WATTLE SIZE
1. Large
2. Small

WATTLE SHAPE
1. Cupped
2. Flat
3. Cupped Flat

SKELETON STRUCTURE
1. Normal
2. Creeper
3. Polydactyl

SEX
1. Male
2. Female

AGRO-ECOLOGICAL ZONE
1. Southern Guinea Savanna
2. Sudano-Sahelian
3. Tropical Rainforest
