# Supplementary material for: Multivariate characterisation of morpho-biometric traits of indigenous helmeted Guinea fowl (Numida meleagris) in Nigeria
Source: PLoS One. 2022 Jun 13;17(6):e0261048. doi: 10.1371/journal.pone.0261048 (PMC9191719; doi:10.1371/journal.pone.0261048)
Supplement: Attachment — Submitted filename: Response to Reviewers.doc [file pone.0261048.s003.doc]

8^th^ March, 2022

The Editor-in-Chief,

PLOS ONE,

USA.

Dear Sir,

**RESPONSE TO REVIEWERS’ COMMENTS ON MANUSCRIPT NO PONE-D-21-36760**

I write to respond to the comments of your highly distinguished reviewers in respect of manuscript no PONE-D-21-36760 entitled ‘Multivariate characterization of morpho-biometric traits of indigenous helmeted guinea fowl (*Numida meleagris*) in Nigeria’. All the comments of the reviewers have been addressed. I do hope that the revised manuscript will be acceptable for publication.

Reviewer #1: Quantitate analyses were done to describe and categorize the Guinea fowl characteristics.

**Comments:** The standard of writing should be re-considered while some of sentences are difficult to understand. This is mostly because of using some strange words like 'veritable', 'embarked'... Please use usual words and phrases instead.

**Response:** We have revisited the whole manuscript for appropriate use of words and also subjected it to professional English editing from Senior Colleagues.

**Comments:** What I am wondering about, after all the analysis, is how this result may help the birds producers and Poultry researchers. I feel that the results are more of interest for Zoologist rather than Commercially poultry community.

**Response:** Thanks for your observation. However, I wish to affirm that the results will be of immense usefulness to Zoologists and the Poultry Community because the first step in the genetic improvement of indigenous stock involves phenotypic characterization. It is a significant tool in breeding programs that allows the preservation of animal biodiversity and supports consumer demands. Similarly, the phenotypic traits can be further explored for improvement, as they provide useful preliminary information for genomic studies.

**Comments:** Different ways for analyses were used. However it is not quite clear what was the point to use all the methods. The results of decision tree is not described appropriately. After each method, there may be a clear paragraph about the precision and accuracy of the models and then conclusion about basing results.

**Response:** The essence of using different statistical analyses is to come up with a categorical statement with respect to the homogeneity or heterogeneity of the indigenous Guinea fowl populations in three different agro-ecological zones in Nigeria. Information that is not revealed by one statistical method may be revealed by the other, and collectively, a more meaningful and valid conclusion can be made. This will guide appropriately the process of selection of birds for genetic improvement. Based on the concern of the reviewer, we consulted a statistical expert on Decision Trees, Assoc. Prof. Şenol Çelik of the Department of Biometrics and Genetics, Faculty of Agriculture, [Bingöl University](https://scholar.google.com.tr/citations?view_op=view_org&hl=tr&org=3464531547582027407), Turkey to bring out the best out of the decision trees. We have provided two additional Tables, one each for CHAID and Exhaustive CHAID to provide information on precision and accuracy including model comparison. We have also described and discussed the results appropriately as suggested.

**Comments:** The references list should be shortened.

**Response:** We have reduced the number of references as suggested.

Reviewer #2

**Comments:** The reviewer read this manuscript repeatedly and carefully, nevertheless could not understand the relationship of morpho-biometric trait of the guinea fowl and "agro-ecology", which are the subjects of this manuscript. The term "agro-ecology" which was used a lot in this manuscript, seems to be less specific and obscure the context of the entire manuscript.

**Response:** We have provided a map of Nigeria to show the three different agro-ecological zones for proper delineation and understanding. We have also described each agro-ecological zone based on its peculiar coordinates, climate and vegetation. This is different from the description provided in the original manuscript where we described study locations in each agro-ecological zone. Our null hypothesis is that the qualitative (phaneroptic) and quantitative (morphometric) traits of Guinea fowls in the three agro-ecological zones are statistically (P>0.05) the same. The alternative hypothesis is that the phaneroptic and morphometric traits are statistically (P<0.05) different.

**Comments:** Materials and methods lack a description of procedures that provide objective reproducibility. Please describe the process concretely for the materials and methods, and organize the results and considerations in the order in which the materials and methods are described. In particular, there is no concrete description necessary to understand how to use statistical software and what analysis results lead to what conclusions.

**Response**: We have provided more information on materials and methods and organized the results and discussion in the order in which the materials and methods were described. We have also expanded the description of the use of the statistical software and linked results to conclusions. The first paragraph of the revised Discussion took care of Descriptive statistics under Materials and methods (Tables 1 and 2 of Results). The second paragraph discussed Correspondence analysis (Figure 2 of Results). The third paragraph dealt with Univariate analysis (Tables 3, 4 and 5 of Results). The fourth paragraph highlighted the Stepwise canonical discriminant analysis (Figure 3, Tables 6 and 7 of Results). The fifth paragraph discussed Categorical principal component analysis (Table 8 and Figure 4 of Results) while the sixth paragraph discussed Decision trees (Figures 5 and 6, and Tables 9 and 10 of Results).

**Comment:** Please unify the terms used in a unique sense.

**Response:** We have unified the terms used in a unique sense as suggested. For instance, we have replaced qualitative traits with ‘qualitative physical traits’ throughout the text. We are also consistent with the use of ‘biometric traits and morphological indices’ to describe the quantitative traits.

**Comment:** The reviewer could not open and view the File of Data 1-Qualitative.

**Response:** We have merged the qualitative traits and the quantitative traits data and provided a single excel file under supplementary information (S1 DATA) in the revised manuscript. We are very sorry that the initial Data 1-Qualitative file could not be opened.

**Comment:** Line 80: This sentence seems to indicate the purpose of the current study, but I don't understand what "qualitative traits" mean. Also, does "liner body measurement" mean measurement of specific body parts? For example, does it mean "The current study aimed to find morphological differences in Nigerian guinea fowl based on specific quantitative indicators."?

**Response:** By "qualitative traits"**,** we mean those physically observable traits in animals that are discontinuous and discrete. Examples are plumage colour, skin colour, helmet shape, etc. Yes, "liner body measurement" means the same thing with biometric traits or measurements. We have recast the sentence as follows: ‘The current study aimed to find differences in indigenous Guinea fowl based on qualitative physical traits, biometric traits and morphological indices in three agro-ecological zones in Nigeria.

**Comment:** Line 95: What does “the relative availability of indigenous” mean objectively in this study? Also, is the "ease of data collection" appropriate as a research design for scientific verification?

**Response:** Guinea fowl distribution in Nigeria is not as widespread as chicken, hence the use of ‘relative availability’. However, we have deleted the sentence together with "ease of data collection" in the course of revision.

**Comment:** Line 115: The reviewer suspected that the following points regarding the sampling may affect the results of the statistical analysis: differences in the number of bird samples extracted from each of the three defined zones, the difference of male-female ratio, the individual willingness to participate. Please explain to get rid of these concerns.

**Response:** Based on the concern of the reviewer, we revisited the original Tables 3, 4, and 5 that reported fixed and interaction effects of the quantitative traits. Due to unequal sample sizes, low male-female ratio and non-normality of the distribution, we decided to test only fixed effect of agro-ecology, sex and sexes within each agro-ecology. For fixed effect of agro-ecology, we used the non-parametric Kruskal-Wallis H test to compare mean ranks of the biometric measurements and morphological indices. Where there were significant (P<0.05) differences, the mean ranks were separated using Dunn-Bonferroni test following the description of Brown et al. (2017). For both sex and sexes within each agro-ecology effects, we equally used non-parametric Kruskal-Wallis H test, but significant (P<0.05) mean ranks were separated using Mann–Whitney U test. We had reflected these changes under statistical analyses, presented and discussed the results appropriately in the revised manuscript. We had earlier reported in the original manuscript the use of Kruskal-Wallis H test for the qualitative physical traits.

**Comment:** Line 116: Please do not omit "zone".

**Response:** We have included "zone".

**Comment:** Line 117: Is "270 birds" correct for "290 birds"

**Response:** The correct figure is 270 birds. We are very sorry for the error.

**Comment:** Line 180: It can be read from the context that the zone division described by the term “the three agro-echological zone” means the three zones shown in lines 94-95. However, for more contextual clarity, the reviewer recommended defining that “the three agro-echological zones” are the three zones shown on lines 94-95.

**Response:** For the purpose of clarity, we have provided the map of the three distinct agro-ecological zones.

**Comment:** Line 194: Please write the CHAID abbreviation first.

**Response:** We have rewritten it as ‘Chi-square automatic interaction detection (CHAID)’.

Reviewer #3

Comments to the Author The manuscript entitled " Multivariate Characterization of Morpho-biometric Traits of Indigenous Helmeted Guinea Fowl (Numida meleagris) in Nigeria" represents a considerable amount of work. The following comments need to be addressed before the manuscript is suitable for publication in Plos One Journal.

**Response:** We thank the reviewer for the positive comment. We have addressed the queries as indicated below:

**Comment:** Line 42: Please change Inspite of to In spite of

**Response:** We have changed ‘Inspite of’ to ‘In spite of’.

**Comment:** Line 117: There is wrong in total number of birds…..290 birds (80 males and 190 females)

**Response:** 290 birds have been changed to 270 birds. We are very sorry for the error.

**Comment:** Line 128: (Guinea fowl) please make the same form for Guinea fowl throughout the manuscript. Because in some parts, you write guinea fowl.

**Response:** We are consistent with the use of Guinea fowl in the revised manuscript.

Every other correction has been effected as suggested. At this juncture, I will like to express my profound gratitude to you, members of the Editorial Board and your highly distinguished reviewers for the time and painstaking efforts.

I look forward to hearing your favourable decision on the manuscript.

.

Yours sincerely,

**Prof. A. Yakubu**
