# Supplementary material for: Multivariate characterisation of morpho-biometric traits of indigenous helmeted Guinea fowl (Numida meleagris) in Nigeria
Source: PLoS One. 2022 Jun 13;17(6):e0261048. doi: 10.1371/journal.pone.0261048 (PMC9191719; doi:10.1371/journal.pone.0261048)
Supplement: Attachment — Submitted filename: Response to Reviewers.doc [file pone.0261048.s004.doc]

3^rd^ May, 2022

The Editor-in-Chief,

PLOS ONE,

USA.

Dear Ma,

**RESPONSE TO REVIEWER’S COMMENTS ON MANUSCRIPT NO PONE-D-21-36760R1**

I write to respond to the comments of your highly distinguished reviewer in respect of manuscript no PONE-D-21-36760R1 entitled ‘Multivariate characterisation of morpho-biometric traits of indigenous helmeted guinea fowl (Numida meleagris) in Nigeria’. All the comments of the reviewer have been addressed. It is my sincere hope that the revised manuscript will be acceptable for publication.

**Academic Editor**

**General Comment:** It needs minor changes for the details of the purpose of the study method in the trial and the answer to the question of which bird communities are useful for further genetic reproduction.

**Response:** We have appropriately responded to the reason for using different algorithms, and also answered the question as regards which bird communities are useful for further genetic reproduction.

**Specific Comments**

**Comment:** A rebuttal letter that responds to each point raised by the academic editor and reviewer(s). You should upload this letter as a separate file labeled 'Response to Reviewers'.

**Response:** A rebuttal letter has been uploaded.

**Comment:** A marked-up copy of your manuscript that highlights changes made to the original version. You should upload this as a separate file labeled 'Revised Manuscript with Track Changes'.

**Response:** A copy of the manuscript that highlights changes made has been appropriately labeled and uploaded.

**Comment:** An unmarked version of your revised paper without tracked changes. You should upload this as a separate file labeled 'Manuscript'.

**Response:** An unmarked version of the revised paper has been uploaded.

**Comment:** in your research, in which you used 74 references, you could also access and add recent papers on guinea fowls that could contribute greatly.

**Response:** As suggested, we have added additional four manuscripts to beef up our work.

**Reviewer** #**1**

**General comments:** My main concern mentioned in first round of review is still exist:

''Different ways for analyses were used. However it is not quite clear what was the point to use all the methods.''.

Simply, in this manuscript, there are several methods of data clustering used for handling a relatively small data set. As you know each of methods produced results for characterizing Guinea fowl. Generalization of the results are limited because we actually do not know about goal of using each methods. Sorry but I can say this is a simple data analysis using several algorithm that produced unclear results.
Answer to the question like which community of birds are useful for further genetic breeding was your priority in this study. So with your results there is no such a clear answer to that question.

**Response:** We highly appreciate the concern of the highly distinguished reviewer as regards the need to make clearer the purpose of using different algorithms, and the inference that can be drawn from the study. We used different algorithms in order to be able to arrive at a more informed decision especially the development of phenotypic standards for breeding and genetic purpose as proposed by FAO (2012). When morphometric traits are considered jointly, multifactorial analyses (using different algorithms) have been shown to assess better the within-population variation which can be utilized in the discrimination of different population types. What we have done is similar to what some of our distinguished peers have published on related topics in poultry species (Traore et al. 2018; Otecko et al., 2019; Toalombo Vargas et al., 2019; Brito et al., 2021; González Ariza, et al., 2021). However, we have included an additional paragraph where we highlighted the general implication of our findings based on the algorithms used. We reported that guinea fowls from the Southern Guinea Savanna, Sudano-Sahelian and Tropical Rainforest zones of Nigeria are more homogeneous than heterogeneous with respect to the investigated qualitative physical traits, biometric traits and morphological indices. This means that elite birds from the three agro-ecological zones are potential candidates for pure breeding or crossbreeding with their more productive exotic counterparts. Meanwhile, we sincerely welcome further suggestions from the distinguished reviewer on how to make the manuscript better.

Every other correction has been effected as suggested. At this juncture, I will like to express my profound gratitude to you, members of the Editorial Board and your highly distinguished reviewer for the time and painstaking efforts.

I look forward to hearing your favourable decision on the manuscript.

.

Yours sincerely,

**Prof. A. Yakubu**
